# Multi-Trait Multi-Environment Genomic Prediction for End-Use Quality Traits in Winter Wheat

**DOI:** 10.3389/fgene.2022.831020

**Published:** 2022-01-31

**Authors:** Karansher S. Sandhu, Shruti Sunil Patil, Meriem Aoun, Arron H. Carter

**Affiliations:** ^1^ Department of Crop and Soil Sciences, Washington State University, Pullman, WA, United States; ^2^ School of Electrical Engineering and Computer Science, Washington State University, Pullman, WA, United States1

**Keywords:** end-use quality, genomic prediction, heritability, machine learning, multi-trait, secondary traits, wheat

## Abstract

Soft white wheat is a wheat class used in foreign and domestic markets to make various end products requiring specific quality attributes. Due to associated cost, time, and amount of seed needed, phenotyping for the end-use quality trait is delayed until later generations. Previously, we explored the potential of using genomic selection (GS) for selecting superior genotypes earlier in the breeding program. Breeders typically measure multiple traits across various locations, and it opens up the avenue for exploring multi-trait–based GS models. This study’s main objective was to explore the potential of using multi-trait GS models for predicting seven different end-use quality traits using cross-validation, independent prediction, and across-location predictions in a wheat breeding program. The population used consisted of 666 soft white wheat genotypes planted for 5 years at two locations in Washington, United States. We optimized and compared the performances of four uni-trait– and multi-trait–based GS models, namely, Bayes B, genomic best linear unbiased prediction (GBLUP), multilayer perceptron (MLP), and random forests. The prediction accuracies for multi-trait GS models were 5.5 and 7.9% superior to uni-trait models for the within-environment and across-location predictions. Multi-trait machine and deep learning models performed superior to GBLUP and Bayes B for across-location predictions, but their advantages diminished when the genotype by environment component was included in the model. The highest improvement in prediction accuracy, that is, 35% was obtained for flour protein content with the multi-trait MLP model. This study showed the potential of using multi-trait–based GS models to enhance prediction accuracy by using information from previously phenotyped traits. It would assist in speeding up the breeding cycle time in a cost-friendly manner.

## Introduction

Wheat (*Triticum aestivum* L.) is one of the most important staple crops worldwide, providing 18% of the caloric intake (Awika, 2011; Saini et al., 2022). Hexaploid wheat is categorized into soft and hard wheat classes based on protein strength, kernel texture, water absorption, and milling quality (Kiszonas et al., 2013). In the United States, six major classes of wheat, namely, hard white wheat, hard red spring wheat, hard red winter wheat, soft white wheat, soft red winter wheat, and durum, are grown in different regions. Soft white wheat (SWW) is a predominant class in eastern Washington and the inland Pacific Northwest (Kiszonas and Morris, 2018). SWW is one of the wheat classes with high demands from overseas markets in countries like the Philippines, Korea, Japan, and Indonesia, due to its high end-use quality. Soft wheat is mainly used for making cakes, cookies, pastries, Asian-style noodles, crackers, and pretzels (Morris et al., 2008). In addition to having high grain yield, disease and insect resistance, wide adaptability, and cold tolerance, the released wheat cultivar needs to maintain high end-use quality attributes required by millers, bakers, and grain markets (Morris et al., 2009; Carter et al., 2012; Guzman et al., 2016; Sandhu et al., 2021d).

Phenotyping for end-use quality traits is usually delayed until advanced generations in wheat breeding owing to the associated cost, labor, and amount of seed required (Battenfield et al., 2016). Delayed phenotyping usually results in hindrance of releasing promising cultivars due to lack of end-use quality data to make decisions. Important end-use quality traits in wheat include cookie diameter, flour sedimentation value, flour yield, grain protein content, and milling score (Campbell et al., 2007; Kiszonas et al., 2015). Linkage and association mapping have been used to identify the genomic loci controlling end-use quality traits, and most of the major effect genes are now fixed into the breeding programs for different market classes (Jernigan et al., 2018; Yang et al., 2020). Marker-assisted selection has been used to screen for some major effect end-use quality genes in wheat classes based on granule-based starch synthase 1, low and high molecular weight glutenins, and kernel texture (Aoun et al., 2021b). These major effect loci only assist in differentiating between different classes but do not provide the complete profile (Kumar et al., 2019). Association mapping studies in wheat have shown that more than 300 small effect QTLs control these end-use quality traits and suggest the quantitative nature of these traits, requiring appropriate strategies to be adopted in breeding programs for selection (Breseghello and Sorrells, 2006; Bhave and Morris, 2008; Jernigan et al., 2018; Yang et al., 2020).

The ultimate interest of a plant breeding program is to enhance the long-term genetic gain, and in modern terms, genetic gain is defined as $\Delta G=i\sigma_{A}r/t$ , where ΔG is the rate of the gain/response to selection, $\sigma_{A}$ is the square root of the standard additive genetic variance, i is the selection intensity, r is the correlation between genotypic and true breeding values, and t is the length of the breeding cycle (Bernardo, 2016; Cobb et al., 2019a; Cobb et al., 2019b). Genomic selection (GS) is the approach adopted by most plant breeding programs, which enhances the rate of genetic gain by estimating breeding values using whole genome-wide markers without phenotyping (Meuwissen et al., 2001). First, the GS model is trained using previous year phenotypic and genotypic data to estimate marker effect and the model’s performance is assessed using various cross-validation approaches. The trained GS model predicts the genomic estimated breeding values of the selection/breeding population (Lorenz et al., 2011; Lorenz, 2013). Since the last decade, increasing the prediction accuracies for GS has been the main focus of research (Kaur et al., 2021). GS performance is affected by the relationship between testing and training set, trait heritability and architecture, population structure, population size, and the statistical model (Herter et al., 2019; Monteverde et al., 2019).

Most genomic selection studies use the uni-trait model, where a single trait is predicted (Qin et al., 2019; Pérez-Rodríguez et al., 2020; Sandhu et al., 2022). However, plant breeders have shifted to multi-trait (MT) GS models that simultaneously predict two or more traits and demonstrate improved accuracy (Calus and Veerkamp, 2011; Sandhu et al., 2021a). MT models use the shared genetic information between the traits using the same set of predictors with the assumption of some structure in the captured output. MT models leverage the correlation between different traits and show a considerable advantage in other domains, such as ecological modeling, weather forecasting, forest management, and data mining (Voyant et al., 2017). MT models using shared genetic information are important for hard/expensive to phenotype traits having low heritability (Juliana et al., 2019). Several studies have demonstrated the improvement of prediction accuracy for a primary trait with the inclusion of a secondary trait into the MT models in wheat. Sandhu et al. (2021a) showed an improvement of 20 and 12% prediction accuracies for grain yield and grain protein content in wheat, respectively, by including correlated spectral reflectance indices into the model as secondary traits in the MT approach. Similarly, Hayes et al. (2017), Lado et al. (2018), and Bhatta et al. (2020) observed the improvement of prediction abilities with MT models over the uni-trait models for end-use quality traits in cereals.

A previous study from our group showed that GS accuracies varied from 0.27 to 0.81 for 14 end-use quality traits using nine different uni-trait models (Sandhu et al., 2021c). Statistical models used for training the uni- or multi-trait GS models play an important role in evaluating performance (Jia and Jannink, 2012). Ridge regression best linear unbiased prediction (rrBLUP) is one of the most frequently used models for quantitative traits assuming normal distribution of marker effects with constant variances (Endelman, 2011). Bayes Cpi uses variable selection, scaled-t distribution to estimate marker effects and assumes different variances for adjusting to the different genetic architecture of the trait (Pérez and De Los Campos, 2014; Montesinos-López et al., 2019a). rrBLUP and Bayes Cpi are known as parametric models as they assume a prior relationship between features and predictors, and this opens up the avenue for the nonparametric machine and deep learning algorithms. Machine learning models such as random forest, ensemble learning, and support vector machines use algorithms that progressively learn the pattern from sample data to make final predictions (Hastie et al., 2009). Deep learning is one of the branches of machine learning focusing on the artificial neural network for model training and predictions. Deep learning models such as generative neural networks, convolutional neural networks, and recurrent neural networks use different combinations of layers and nonlinear activation functions to transform the data at each layer to obtain a better fit for each trait by considering genetic architecture (Lecun et al., 2015).

In previous studies, we have shown the advantages of multi-trait GS models (Sandhu et al., 2021e) and machine and deep learning models for predicting complex traits in wheat (Sandhu et al., 2021b; Sandhu et al., 2021c). Building upon the findings of previous studies, this study’s objectives were to 1) optimize the uni- and multi-trait GS models for seven end-use quality traits, 2) compare the performances of four uni- and multi-trait GS models using cross-validation and independent predictions, and 3) assess the potential of across-location prediction using multi-trait models and with the inclusion of genotype by environment interaction component.

## Materials and Methods

**Plant material:** A total of 666 SWW genotypes from the Washington State University winter wheat breeding program were screened at two locations, namely, Lind and Pullman, WA, United States, from 2015 to 2019. These genotypes consist of preliminary and advanced yield lines, doubled haploid lines, and F_3:5_ lines screened as part of the breeding program. Genotypes in the advanced and preliminary yield trials were screened for yield, and superior lines were later evaluated for end-use quality traits. Double haploid lines and F_3:5_ derived lines were screened for disease resistance and agronomic traits, and the selected genotypes were screened for quality traits and not for yield traits. As the dataset was from a breeding program, some lines were continuously removed each year with new genotypes in the subsequent year, resulting in an unbalanced dataset. More information about the dataset is referred to Aoun et al., 2021a and Sandhu et al., 2021b. End-use quality data were collected separately at both locations for all the genotypes.

**Phenotyping for the end-use quality traits:** These genotypes were tested for seven end-use quality traits, namely, cookie diameter (CODI), grain protein content (GPC), flour yield (FYELD), flour SDS sedimentation (FSDS), flour ash (FASH), flour protein (FPROT), and milling score (MSCOR). Complete information about all these traits and their summary is provided in Table 1. To evaluate grain characteristics, GPC was measured following AACC Approved Method 39–10.01 using an NIR analyzer (Perten Elmer, Sweden). Flour parameters, namely, FASH, FPROT, and FSD were measured using the extracted flour. FASH and FPROT were measured using Approved methods 08–01.01 and 39–11.01. The milling traits, that is, FYELD and MSCOR were measured using the sample obtained from the modified Quadrumat Senior Experimental Milling System. FYELD was estimated as a ratio of total flour by weight (reduction rolls and break). MSCOR was obtained using FYELD and FASH. CODI is one of the baking parameters and is estimated by following the AACC Approved Method 10–52.02. More information about the phenotyping is referred to Aoun et al., 2021a and Sandhu et al., 2021b.

**TABLE 1:** Summary statistics of seven end-use quality traits evaluated from the SWW population.

Trait	Abbreviation	Mean	Standard error	Heritability	Units
Grain protein content	GPC	10.73	0.05	0.56	percent
Flour protein	FPROT	8.93	0.04	0.57	percent
Flour ash	FASH	0.39	0.001	0.88	percent
Milling score	MSCOR	85.6	0.10	0.81	unitless
Flour yield	FYELD	69.9	0.09	0.91	percent
Cookie diameter	CODI	9.2	0.008	0.89	cm
Flour SDS sedimentation	FSDS	10.1	0.09	0.92	g/mL

**Genotyping:** Genotyping by sequencing (GBS) was used for genotyping the complete population using the facilities from Genomics Sciences Laboratory, Raleigh, NC (Poland et al., 2012). The complete details about the genotyping and SNP calling was reported in Aoun et al. (2021a) and Sandhu et al. (2021a). Initial SNP data consisted of 216,392 markers anchored to the *T. aestivum* RefSeq v1.0 reference genome. Markers were removed based on the minor allele frequency less than 5%, heterozygosity more than 15%, and markers missing more than 20% of data, and the whole pipeline was implemented in R (R Development Core Team, 2020). At the end of the filtering, we were left with 40,518 SNPs used for further analysis.

**Phenotypic data analysis:** To account for the unbalanced dataset in this study, adjusted means were extracted using residuals obtained from the unreplicated genotypes in individual environments using the augmented complete block design model implemented in the R statistical program. Adjusted means were obtained according to the method implemented in Sandhu et al. (2021b), and the model equation is given as follows:

$${\text{Y}}_{\text{ij}}={\text{Block}}_{\text{i}}+{\text{Check}}_{\text{j}}+{\text{e}}_{\text{ij}},$$

where Y_ij_ is the raw phenotype, Block_i_ corresponds to the fixed block effect, Check_j_ is the replicated check cultivar effect; Block_i_ is the fixed block effect, and e_ij_ is the residuals.

Adjusted means across the environments were obtained using the models and are given as follows:

$${\text{Y}}_{\text{ijk}}=µ+{\text{Check}}_{\text{i}}+{\text{Block}}_{\text{j}}+{\text{Env}}_{\text{k}}+{\text{Check}}_{\text{j}}\times {\text{Env}}_{\text{k}}+{\text{Block}}_{\text{i}}\times {\text{Env}}_{\text{k}}+{\text{e}}_{\text{ijk}},$$

where Y_ijk_ is the raw phenotype value; Check_j_, Block_i_, and Env_k_ are the fixed effect of the *i*th check, *j*th block, and *k*th environment, respectively; and e_ijk_ is the residuals.

Heritability of each trait was calculated using the model as follows:

$$H_{C}^{2}=1-\frac{{\bar{v}}_{\Delta ..}^{\text{BLUP}}}{2\sigma_{g}^{\wedge 2}},$$

where $H_{C}^{2}$ is the Cullis heritability, ${\bar{v}}_{\Delta ..}^{BLUP}$ is the mean–variance of BLUPs, and $\sigma_{g}^{\wedge 2}\;$ is genotypic variance.

Genetic correlation among traits was obtained using the multivariate models as follows:

$$\left[\begin{array}{c} y_{A}\\ y_{B} \end{array}\right]=\left[\begin{array}{cc} X_{A} & 0\\ 0 & X_{B} \end{array}\right]\left[\begin{array}{c} b_{A}\\ b_{B} \end{array}\right]+\left[\begin{array}{cc} Z_{A} & 0\\ 0 & Z_{B} \end{array}\right]\left[\begin{array}{c} g_{A}\\ g_{B} \end{array}\right]+\left[\begin{array}{c} \varepsilon_{A}\\ \varepsilon_{B} \end{array}\right],$$

where *y*_*A*_ and *y*_*B*_ are the BLUPs of the two traits, X and Z denote the design matrix, g is the random genetic effects, and e is the residual for each trait. Variance components were calculated assuming $\left[\begin{array}{c} g_{A}\\ g_{B} \end{array}\right]$ ∼ *N(0, H⊗G)*, where *H* is the genetic variance–covariance matrix, *G* is the genomic relationship matrix, and $\left[\begin{array}{c} \varepsilon_{A}\\ \varepsilon_{B} \end{array}\right]$ ∼ *N(0, I⊗R)*, where *I* is the identity matrix and *R* is the residual variance–covariance matrix. The genetic correlation is calculated as follows:

$$r_{G}=\frac{\mathrm{cov}\left(A,B\right)}{\sqrt{\mathrm{var}\left(A\right)\cdot \mathrm{var}\left(B\right)}},$$

where *cov(A, B)* is the covariance between two traits, Var*(A)* and Var*(B)* represent variances of two traits individually, and the analysis was performed using JMP genomics (SAS Institute Inc, 2011).

**Genomic selection models:** We evaluated the performances of four uni-trait and multi-trait GS models for predicting seven end-use quality traits, and prediction accuracy was compared under different validation scenarios to mimic the breeding program. These four models were GBLUP, Bayes B, RF, and MLP and were tried under both uni-trait and multi-trait scenarios. Complete information about the model structure and optimization is provided below:

**Genomic best linear unbiased predictor:** The uni-trait GBLUP model was used to train each trait individually, and the model is represented as follows:

y=µ+Zu+e,

where y is the vector of end-use quality phenotype for each genotype, µ is the overall mean, u is a vector of normally distributed marker predictor effects as u ∼ N (0, I $\sigma^{2}$ _u_), Z is a design matrix assigning markers to genotypes, and e is the residual error with e ∼ N (0, I $\sigma^{2}$ _e_). The multi-trait model is represented as follows:

$$\left[\begin{array}{c} y_{1}\\ .\\ .\\ .\\ yn \end{array}\right]=\left[\begin{array}{cc} X & 0\\ . & .\\ . & .\\ . & .\\ 0 & X_{n} \end{array}\right]\left[\begin{array}{c} {µ}_{1}\\ .\\ .\\ .\\ {µ}_{n} \end{array}\right]+\left[\begin{array}{cc} Z & 0\\ . & .\\ . & .\\ . & .\\ 0 & Z_{n} \end{array}\right]\left[\begin{array}{c} u_{1}\\ .\\ .\\ .\\ u_{n} \end{array}\right]+\left[\begin{array}{c} \varepsilon_{1}\\ .\\ .\\ .\\ \varepsilon_{n} \end{array}\right],$$

where n is the number of traits, y _1 to n_ represents the vector of phenotypes of the end-use quality traits, X and Z are design matrix, and $\left[\begin{array}{c} u_{1}\\ .\\ .\\ .\\ u_{n} \end{array}\right]$ represents the random marker effects, distributed as ∼ N (0, G⊗H), where G is the genomic relationship matrix, H is the variance–covariance matrix, and $\varepsilon_{1\ldots n}$ represents the standard normal error, distributed as ∼ N (0, I⊗R), where R is the residual variance–covariance matrix and I is identify matrix.

**Bayesian B:** The uni-trait Bayes B model was used to train each trait individually, and the model is represented as follows:

$$y_{i}=µ+\overset{j=p}{\underset{j=1}{\sum }}x_{ij}\beta_{j}+\varepsilon_{i},$$

where $y_{i}$ is the vector of end-use quality phenotype for each line, $x_{ij}$ is the identity of the SNP, $\beta_{j}$ represents the marker effect, µ is the overall mean, and $\varepsilon_{i}\;$ is residual error. MTM and BGLR packages were used for the analysis with 5,000 burn-in and 15,000 test iterations (de los Campos and Grüneberg, 2016). Prior distribution used for model training is as follows:

$$\beta_{j}|\sigma_{j}^{2},\;\Pi =\{\frac{0\;\;with\;probability\;\pi }{N\left(0,\sigma_{j}^{2}\right)\;with\;probability\;1-\pi }\;,$$

which is a mixture of distribution with mass at zero and same prior for all remaining markers, that is, *χ* ^*−2*^ (*df*_*β*_ *, S*_*β*_) where *S*_*β*_ is a scaling parameter and *df*_*β*_ is the degree of freedom (Pérez and De Los Campos, 2014).

The MT Bayes B model is represented as follows:

y=µ+Zu+ε,

where *y* represents the vector of phenotypes of the end-use quality traits, µ is the overall mean, u is the genotypic value distributed as u *∼ N(0, H⊗G)*, and ε is residuals.

**Bayesian multi-trait multi-environment model (BMTME):** Montesinos-López et al., 2016, Montesinos-López et al., 2019b provided a BMTME model for predictions which is represented as follows:

$$y=X\beta +Z_{1}b_{1}+Z_{2}b_{2}+\varepsilon ,$$

where y is the matrix of order t x l, with t is the number of traits and l = e x g, where g is the number of genotypes and e is the number of environments; X, Z1, and Z2 are design matrixes for environmental effect, genotypic effect, and genotype by environmental interaction, respectively; β is beta coefficient matrix of order e x t; $b_{1}$ is the random genotypic effect distributed as $b_{1}$ *∼ MN*(*0, G, Ʃ*_*t*_), where G is additive relationship matrix and *Ʃ*_*t*_ is the unstructured covariance matrix of order t x t; $b_{2}$ is the random genotypic x trait x environment effect matrix distributed as $b_{2}$ *∼ MN*(*0, Ʃ*_*e*_ *G, Ʃ*_*t*_), where *Ʃ*_*e*_ is the unstructured covariance matrix of order e x e. BMTME package was used for the analysis with 5,000 burn-in and 15,000 test iterations (Montesinos-López et al., 2019b).

**Random forests:** RF is a tree-based machine learning model where output is predicted from the collection of identically distributed trees. Input features are split at each node of the tree to create a new branch, and splitting is performed by lowering the loss function. Bootstrap sampling was performed over the training set to select the best set of features for tree building (Ramzan et al., 2020). The model equation is given as follows:

$${\hat{y}}_{i}=\frac{1}{B}\overset{B}{\underset{b=1}{\sum }}T_{b}\left(x_{i}\right),$$

where ${\hat{y}}_{i}$ is the predicted value of the end-use quality trait with genotype $x_{i}$ , T represents the number of trees, and *B* is the number of bootstrap samples. The outline of model optimization is as follows.

1) Bootstrap sampling was performed to select the plants from the training set with replacement and was repeated for b = (1,…, B) times.
2) Max feature (max_feature) function from the random forest regressor library was used to identify the best set of features (SNP) by lowering the loss function while building new trees.
3) Splitting at each node of the tree was performed using genotypic data to lower the mean square error
4) The aforementioned three steps were repeated until a minimum node or maximum depth was reached. The set of these trees were used to predict the output of a genotype $x_{i}$ by averaging the performance over the forest.

The hyperparameter space was explored using the grid search cross-validation (CV) function to optimize the hyperparameters for each trait by lowering the mean squared error. The important hyperparameters used for RF training were number and depth of trees, feature importance, and number of features sampled for each iteration. Hyperparameters tried were number of trees (200, 300, 500, and 1,000), max features (auto and sqrt), and max depth (40, 60, 80, and 100) using random forest regression and Scikit learn libraries.

**Multilayer perceptron (MLP):** MLP is a special type of neural network where information flows in one direction, starting from input layer through different hidden (processing) layers to the output layer. The output from the last hidden layer is used to predict output and is represented as follows:

$$Y_{j}=b_{\left(j-1\right)}+W_{j}f_{\left(j-1\right)}\left(x\right),$$

where *Y*_*j*_ is the output from the *jth* hidden layer, f _*(j-1)*_ is the activation function, *W*_*j*_ is the neuron’s weight, and *b*_*(j-1)*_ is the bias associated with each layer. The number of vectors in the output layer define the uni- and multi-trait models.

The hyperparameter space was explored using the Keras inner grid search cross-validation (CV) function to optimize the hyperparameters for each trait by lowering the mean squared error. For hyperparameter optimization, 80% of the training data were used, where 80% of this dataset was used for exploring the hyperparameter space and the remaining 20% for validation. Scikit learn and Keras libraries were used to optimize the model in Python (Gulli and Pal 2017). A full-factor design was implemented using grid search CV to explore parameters, that is, solvers, dropout, learning rate, number of filters, activation function, number of hidden layers and neurons, and regularizations. Overfitting in the model was controlled using early stopping, regularization, and dropout (Srivastava et al., 2014). More information about the MLP models, hyperparameter optimization, and overfitting control is used in Sandhu et al., 2021c, Sandhu et al., 2021a.

**Assessing the model’s prediction abilities:** The genomic selection model performance was evaluated as prediction accuracy, which is the correlation between GEBVs and the observed phenotype. The correlate function from the “corrr” R package was used to assess prediction accuracy (Max et al., 2020). Cross-validation approach, that is, a five-fold CV was used to evaluate the prediction accuracies where each fold was used separately as a testing fold, and this process was repeated two hundred times. For each location, that is, Pullman and Lind, performances of both uni- and multi-trait models were evaluated separately using five-fold CV, and the results were reported separately for each trait and model.

Across-location prediction scenarios were also tested where the dataset from one location was used to predict the performances of genotypes at another location and environment. In our case, the complete data set from one location, that is, Lind was used to train the model, and predictions were made for 2019 Pullman environment and vice versa. Genotype by environment components was also included during across-location predictions.

## Results

**FIGURE 1:**
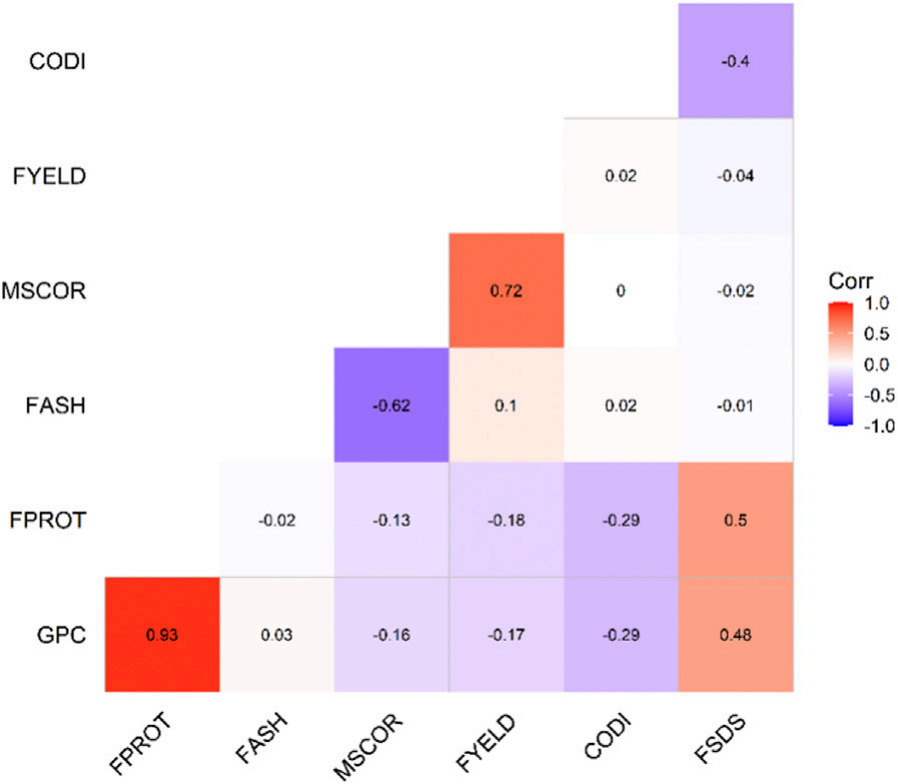
Phenotypic correlation among the seven end-use quality traits evaluated from the SWW population.

**FIGURE 2:**
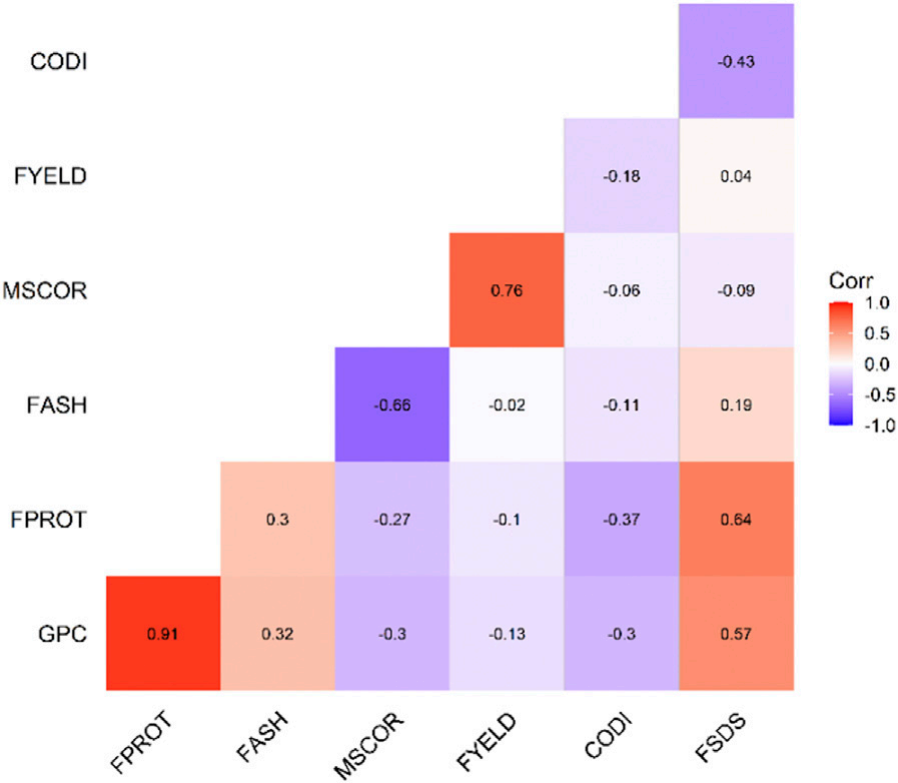
Genetic correlation among the seven end-use quality traits evaluated from the SWW population.

**FIGURE 3:**
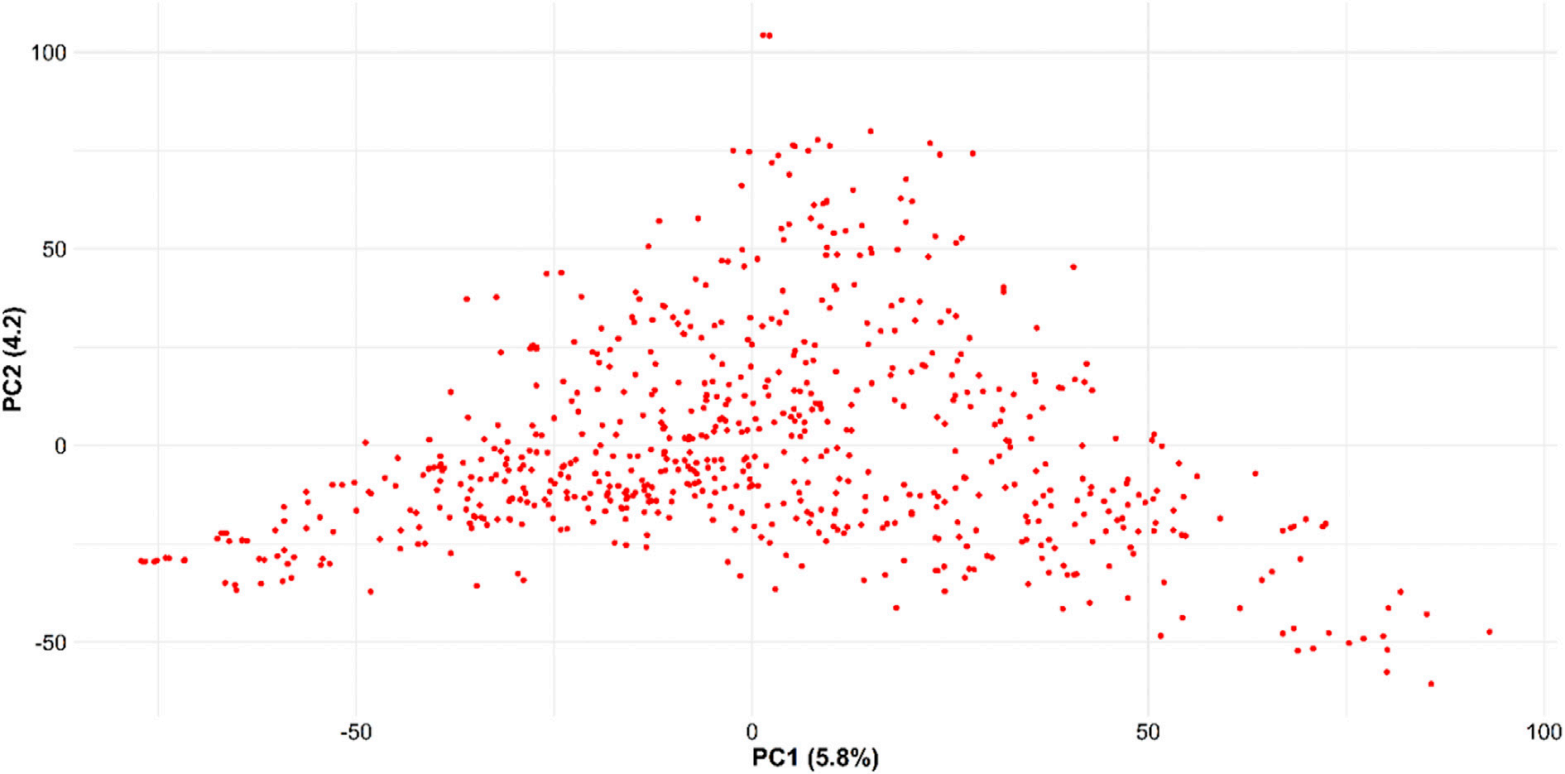
Principal component analysis for the 666 SWW genotypes obtained using 40,518 SNP markers.

**Trait summary, heritability, and correlation:** Table 1 provides the summary and broad-sense heritability of seven end-use quality traits evaluated from the SWW population planted at two locations in this study. Most of the traits had moderate to high heritability, except grain protein content and flour protein. Heritability of FSDS and FYELD was 0.92 and 0.91, respectively, highest among all the traits. Phenotypic and genetic correlation results provided evidence that few traits were correlated (Figures 1, 2). The highest phenotypic and genetic correlations were observed between GPC and FPROT, which was 0.93 and 0.91, respectively (Figures 1, 2). Some traits were negatively correlated with each other. Principal component analysis showed the absence of structure in the population, where first and second PCs only explained the 5.8 and 4.2% variation, respectively (Figure 3), and this was expected as the population was from the same breeding program. Frequency distribution for all the traits at both locations is shown in Supplementary Figure S1. Furthermore, ANOVA results showed that all the traits, except CODI, have significant GXE interaction (Supplementary Table S1).

**Hyperparameter optimization for the MLP model:** Two hundred iterations were performed for the MLP model using Keras inner grid search CV function to optimize the hyperparameters for each trait by lowering the mean squared error. The hyperparameters optimized for each trait were later used for predicting traits in the testing set. Tables 2, 3 provide the set of hyperparameters optimized for each trait under the uni- and multi-trait MLP model. Regularization and dropout were used in the model to control the overfitting following Srivastava et al. (2014). The number of hidden layers and neurons played a critical role during model optimization compared to other hyperparameters. For the uni-trait MLP model, some traits required different activation functions other than relu, while for multi-trait MLP, all the traits gave the lowest MSE with a relu activation function. Information about the hyperparameters is provided separately for each trait, demonstrating that different genetic architecture required specific combinations of hyperparameters for best performance (Tables 2, 3).

**TABLE 2:** Hyperparameters optimized for seven end-use quality traits using the uni-trait MLP model.

Hyperparameter	GPC	FPROT	FASH	MSCOR	FYELD	CODI	FSDS
Activation function	relu	relu	tanh	relu	relu	tanh	tanh
Epochs	200	200	100	150	150	200	150
Dropout	0.2	0.2	0.2	0.2	0.2	0.2	0.2
Learning rate	adaptive	adaptive	constant	adaptive	constant	adaptive	constant
No. of hidden layers	4	3	4	3	3	4	2
No. of neurons	(30, 30, 30, 30)	(24, 24, 24)	(50, 50, 25, 25)	(30, 30, 10)	(90, 90)	(100, 50, 25, 25)	(50, 50)
Regularization	0.1	0.1	0.05	0.02	0.05	0.1	0.001
Solver	Adam	Adam	SGD	L-BFGS	SGD	L-BFGS	SGD

**TABLE 3:** Hyperparameters optimized for seven end-use quality traits using the multi-trait MLP model.

Hyperparameter	GPC and FPROT	FPROT and FSDS	FASH and MSCOR	FYELD and MSCOR	CODI and FSDS
Activation function	relu	relu	relu	relu	relu
Epochs	200	200	200	200	200
Dropout	0.2	0.2	0.2	0.2	0.2
Learning rate	adaptive	adaptive	adaptive	adaptive	adaptive
No. of hidden layers	5	4	5	4	4
No. of neurons	(90, 90, 90, 90, 90)	(100, 60, 60, 60)	(50, 50, 50, 50)	(30, 15, 15, 10)	(100, 90, 90, 70)
Regularization	0.1	0.1	0.1	0.1	0.1
Solver	Adam	Adam	Adam	Adam	Adam

**FIGURE 4:**
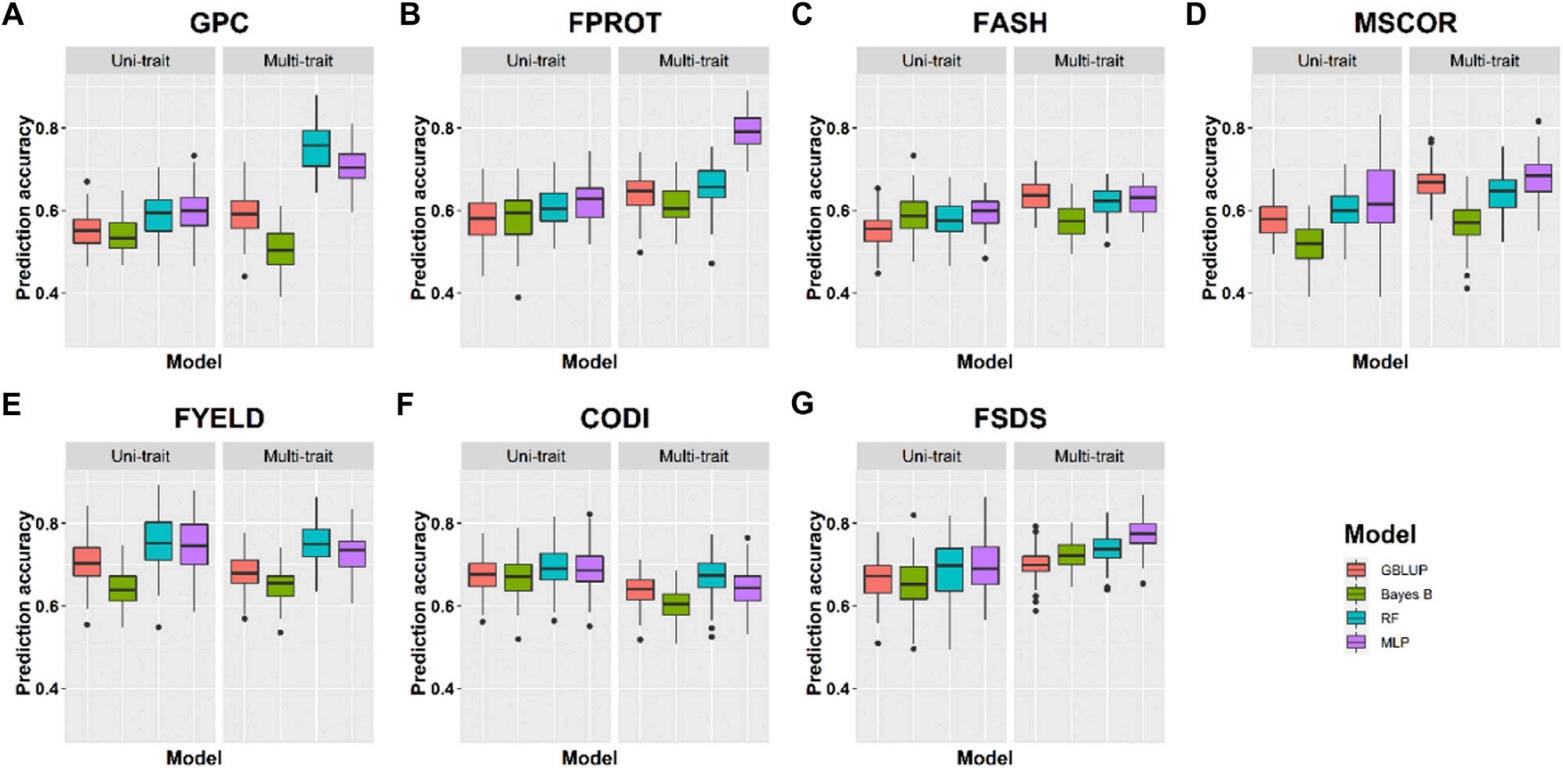
Prediction accuracies for seven end-use quality traits using four different uni- and multi-trait genomic selection models for the Pullman location.

**FIGURE 5:**
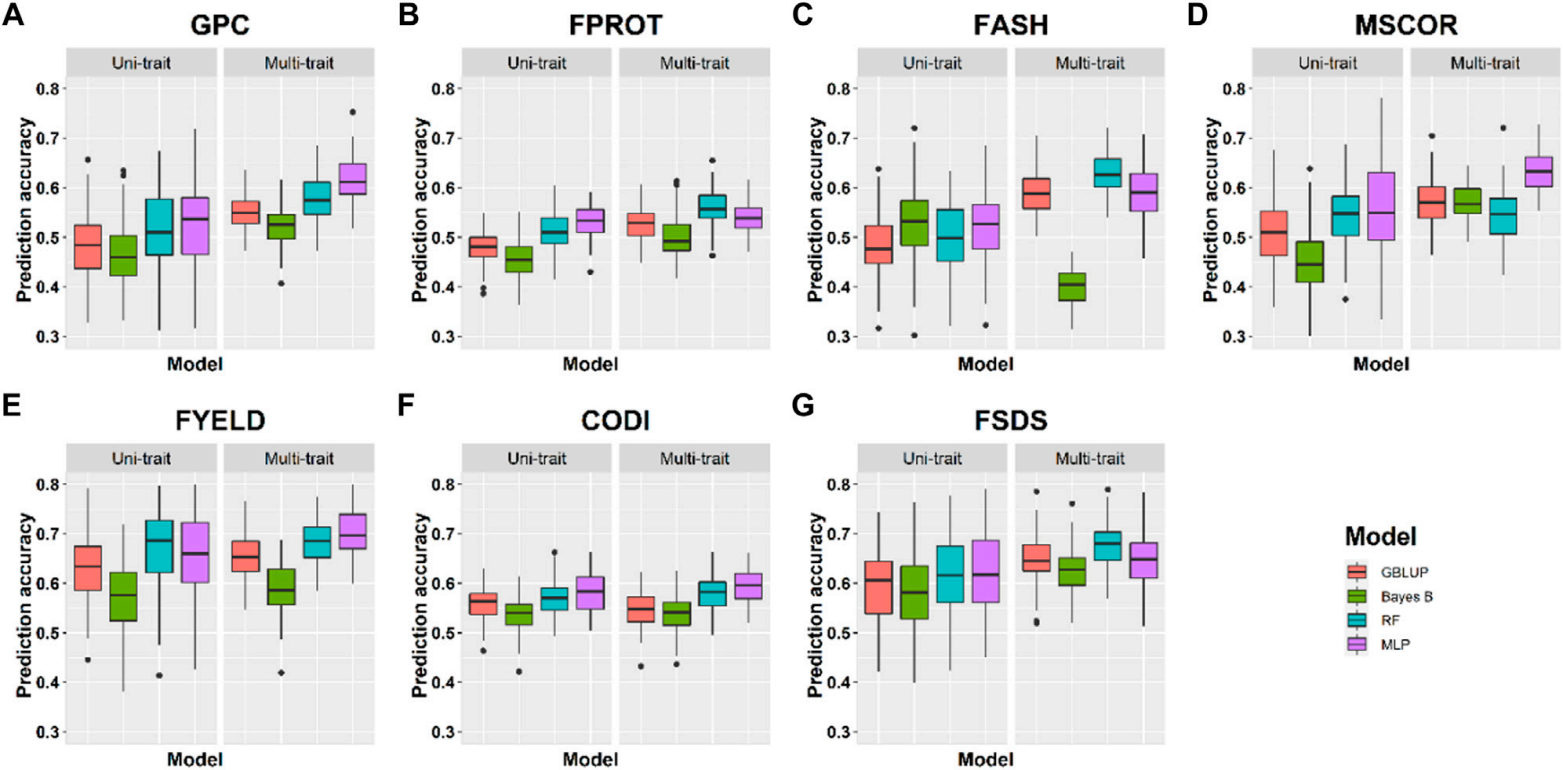
Prediction accuracies for seven end-use quality traits using four different uni- and multi-trait genomic selection models for the Lind location.

**Prediction accuracies within the location using cross-validation:** We compared the performance of four uni- and multi-trait models using a five-fold CV approach to predict seven quality traits. Average results for each trait in the multi-trait GS were used to compare its performance with uni-trait GS models. Figures 4, 5 show the uni- and multi-trait prediction accuracies for the two locations, namely, Pullman and Lind, respectively. Multi-trait prediction accuracies were higher for all the traits, except CODI, for both locations (Table 4). Prediction accuracies varied from 0.44 to 0.76 and from 0.40 to 0.79 for uni- and multi-trait models, respectively, for seven traits evaluated in this study (Figures 4, 5). The Bayes B uni-trait model obtained the lowest prediction accuracies, while the MLP multi-trait model obtained the highest prediction accuracies. On average, multi-trait GS models gave 5.5% higher prediction accuracies than uni-trait GS models (Table 4). There was no difference in the uni- and multi-trait Bayes B model’s performance for most traits. In summary, multi-trait GBLUP, Bayes B, RF, and MLP performed 6.9, 1.8, 6.6, and 6.5% superior to their uni-trait counterparts, respectively (Table 4).

**TABLE 4:** Prediction accuracies for seven end-use quality traits using four different uni- and multi-trait genomic selection models for the two locations across the years, namely, Pullman and Lind using the cross-validation approach.

		Uni-trait models				Multi-trait models			
**Location**	**Trait**	**GBLUP**	**BayesB**	**RF**	**MLP**	**GBLUP**	**BayesB**	**RF**	**MLP**
Pullman	GPC	0.55	0.54	0.59	0.60	0.59	0.50	**0.76**	0.72
	FPROT	0.58	0.58	0.61	0.62	0.64	0.61	0.66	**0.79**
	FASH	0.55	0.59	0.58	0.59	0.63	0.58	0.62	**0.63**
	MSCOR	0.58	0.52	0.60	0.63	0.66	0.57	0.64	**0.68**
	FYELD	0.71	0.64	**0.76**	0.75	0.68	0.65	0.75	0.73
	CODI	0.67	0.67	**0.69**	**0.69**	0.64	0.61	0.67	0.64
	FSDS	0.67	0.66	0.69	0.70	0.71	0.72	0.73	**0.77**
									
Lind	GPC	0.51	0.51	0.54	0.55	0.55	0.53	0.58	**0.62**
	FPROT	0.48	0.46	0.51	0.53	0.53	0.50	**0.56**	0.54
	FASH	0.51	0.44	0.54	0.56	0.59	0.40	0.62	**0.60**
	MSCOR	0.48	0.53	0.50	0.52	0.57	0.57	0.55	**0.63**
	FYELD	0.64	0.58	0.68	0.67	0.66	0.59	0.69	**0.70**
	CODI	0.56	0.54	0.57	0.58	0.55	0.54	0.58	**0.59**
	FSDS	0.59	0.59	0.62	0.63	0.64	0.62	**0.67**	0.64
**Average**		**0.58**	**0.56**	**0.61**	**0.62**	**0.62**	**0.57**	**0.65**	**0.66**

Highest prediction accuracies are bolded for each trait.

The highest prediction accuracies were obtained using a multi-trait MLP model for five of the seven traits evaluated in this study, closely followed by the multi-trait–based RF and GBLUP model. FPROT showed the greatest improvement in prediction accuracy, that is, 36%, with the multi-trait model compared to uni-trait GS models, while CODI showed the lowest improvement in prediction accuracy, that is, -2.9%. Prediction accuracies for the Pullman and Lind locations varied from 0.52 to 0.79 and from 0.40 to 0.70, respectively, with higher accuracy for all the traits at the Pullman location. Improvement in prediction accuracies for GPC, FASH, MSCOR, FYELD, and FSDS with multi-trait models was -0.1–31.6%, 5.4–15.4%, 9.6–31.6%, 1.5–2.3%, and 7.6–16.7%, respectively (Figures 4, 5).

**FIGURE 6:**
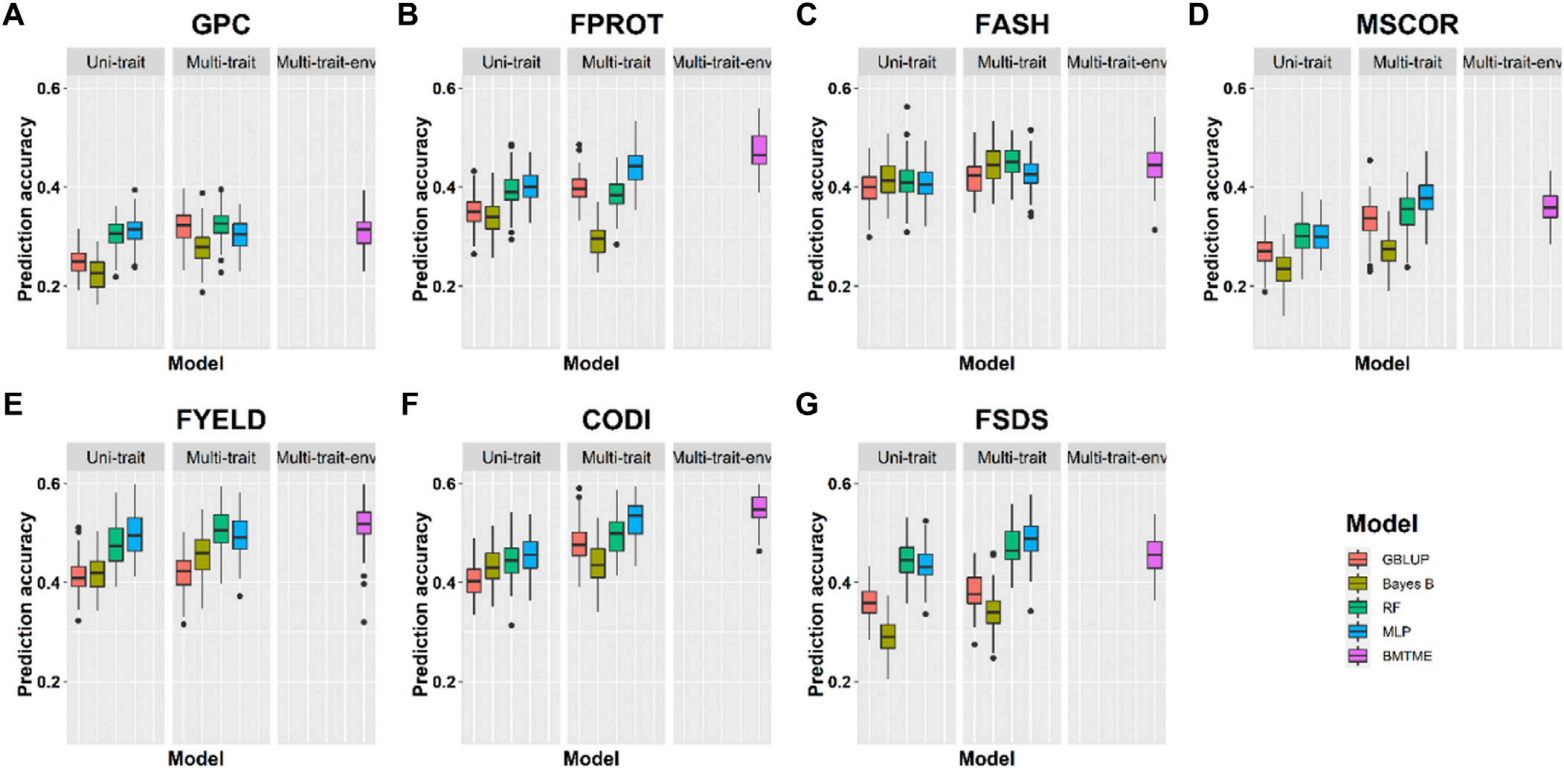
Prediction accuracies across environment Pullman with training on the Lind dataset for seven end-use quality traits using four different uni- and multi-trait and one Bayesian multi-trait multi-environment genomic prediction models.

**FIGURE 7:**
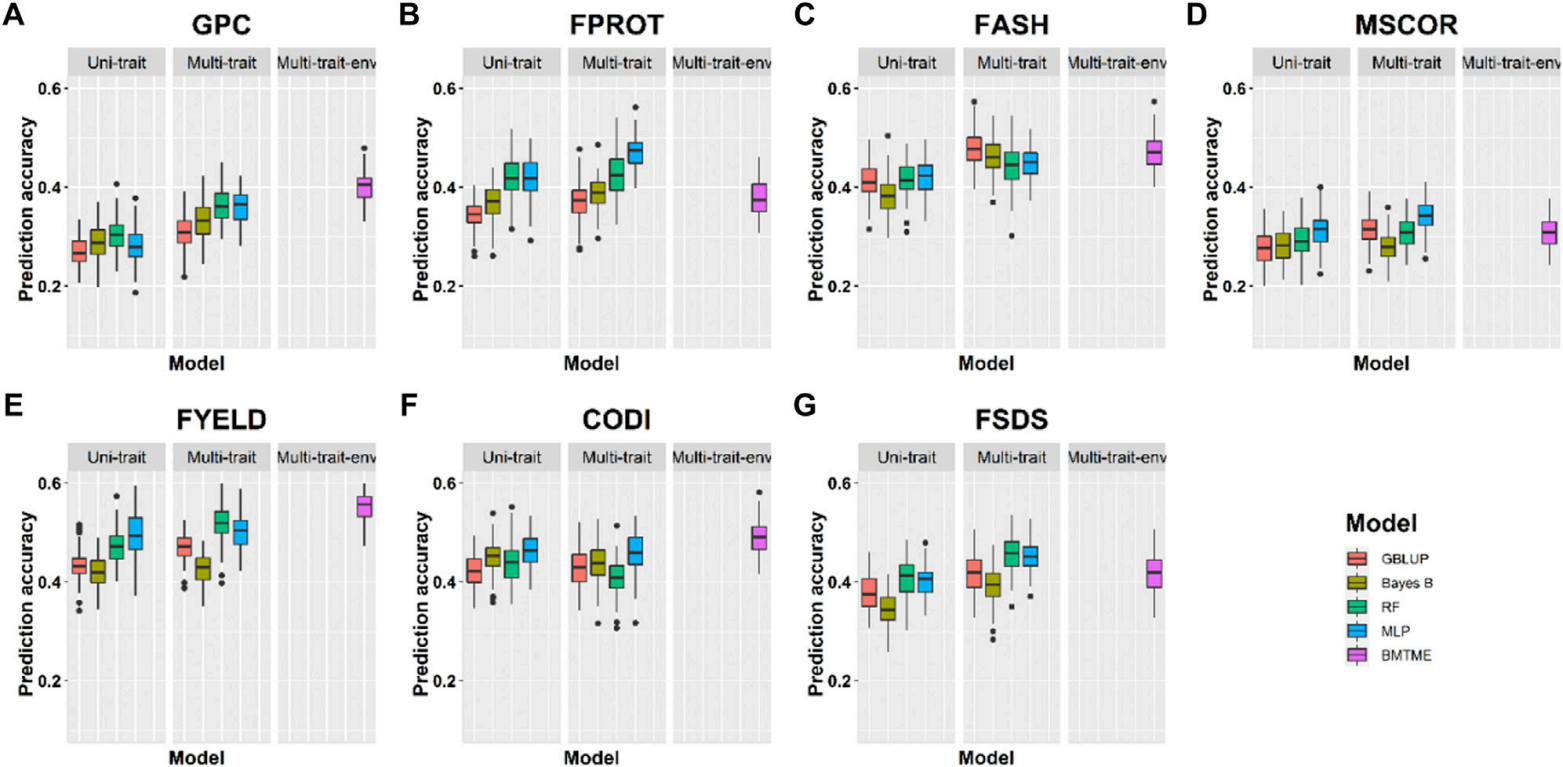
Prediction accuracies across environment Lind with training on the Pullman dataset for seven end-use quality traits using four different uni- and multi-trait and one Bayesian multi-trait multi-environment genomic prediction models.

Prediction accuracies across the environments: Across-location predictions were performed where data from the Pullman environment was used for model training and predictions were made for the Lind environment, and vice versa. Across-location prediction accuracies were lower than prediction accuracies within the environment using cross-validation (Tables 4, 5). Figure 6 and Table 5 show the prediction accuracies for 2019_Pullman when the model was trained on Lind data, and predictions were made for seven end-use quality traits with four different uni- and multi-trait GS and one multi-trait multi-environment model. Similarly, Figure 7 and Table 5 show the prediction accuracies for 2019_Lind when the model was trained using the Pullman dataset. Across-location prediction accuracies varied from 0.25–0.50, 0.28–0.48, to 0.31–0.56 for uni-trait, multi-trait, and multi-trait multi-environment models, respectively, for seven traits evaluated in this study. Similar to cross-validation results, Bayes B models performed inferior compared to all other models.

**TABLE 5:** Prediction accuracies for seven end-use quality traits using four different uni- and multi-trait genomic prediction models for the across-location predictions. 2019_Pullman_Lind represents the scenario where predictions were made on 2019_Pullman by training models on the Lind dataset.

		Uni-trait models				Multi-trait models				Multi-trait multi-environment models
**Location**	**Trait**	**GBLUP**	**BayesB**	**RF**	**MLP**	**GBLUP**	**BayesB**	**RF**	**MLP**	**BMTME**
2019_Pullman_Lind	GPC	0.25	0.23	0.30	0.31	0.32	0.28	**0.33**	0.31	0.31
	FPROT	0.35	0.34	0.40	0.40	0.40	0.29	0.39	0.44	**0.47**
	FASH	0.40	0.41	0.41	0.41	0.42	**0.45**	0.44	0.43	**0.45**
	MSCOR	0.27	0.23	0.30	0.30	0.33	0.27	0.35	**0.38**	0.36
	FYELD	0.41	0.42	0.48	0.50	0.42	0.45	0.51	0.50	**0.52**
	CODI	0.40	0.43	0.45	0.46	0.47	0.44	0.49	0.53	**0.56**
	FSDS	0.36	0.30	0.44	0.43	0.38	0.34	0.47	**0.48**	0.46
										
2019_Lind_Pullman	GPC	0.27	0.29	0.30	0.28	0.31	0.33	0.37	0.36	**0.40**
	FPROT	0.34	0.37	0.42	0.42	0.37	0.39	0.42	**0.47**	0.38
	FASH	0.41	0.38	0.42	0.42	**0.48**	0.46	0.44	0.45	0.47
	MSCOR	0.28	0.28	0.29	0.31	0.31	0.28	0.31	**0.34**	0.31
	FYELD	0.43	0.42	0.47	0.50	0.47	0.43	0.52	0.51	**0.55**
	CODI	0.42	0.45	0.44	**0.46**	0.43	0.44	0.41	0.46	**0.49**
	FSDS	0.38	0.35	0.41	0.40	0.42	0.39	**0.45**	**0.45**	0.42
**Average**		**0.37**	**0.35**	**0.40**	**0.40**	**0.40**	**0.37**	**0.42**	**0.44**	**0.42**

Highest prediction accuracies are bolded for each trait.

We observed that multi-trait GS models performed 7.9% superior compared to uni-trait GS models, and it further strengthens the results obtained for within the environment scenario that multi-trait GS models are better for predicting end-use quality traits. Multi-trait GBLUP, Bayes B, RF, and MLP performed 8.1, 5.7, 5.0, and 10.0% superior to their uni-trait counterparts, respectively (Table 5). Improvement in prediction accuracies for GPC, FPROT, FASH, MSCOR, FYELD, CODI, and FSDS with multi-trait models was 21.7–43.4%, -14.7–29.4%, 5.0–12.5%, -17.4–65.2%, 2.4–24.4%, 10.0–32.5%, and 13.3–60.0%, respectively, over the uni-trait models (Figures 4, 5). There was no difference in the performance of multi-trait machine and deep learning models from the multi-trait multi-environment model which consisted of genotype by environmental interaction in the model (Table 5).

## Discussion

Plant breeders routinely collect data for multiple traits from multiple environments before making final selections. Genomic selection is becoming popular to predict GEBVs due to robust next-generation sequencing technologies and its cost-effectiveness. However, few studies have utilized the multi-trait and multi-environment prediction models due to the model’s complexity, huge computational burden, and lack of good quality phenotyping data (Cuevas et al., 2017). Multi-environment prediction represents a perfect scenario to reduce the number of locations or plots needed in subsequent selection trials (Tolhurst et al., 2019; de Oliveira et al., 2020). Multi-trait GS models showed improved prediction accuracy in previous studies when traits are correlated and have low heritability; these models provide an opportunity to predict traits simultaneously by borrowing information from each other (Gill et al., 2021; Larkin et al., 2021). This study explored the potential of using multi-trait–based GS models to predict seven end-use quality traits in soft white wheat population planted at two locations in Washington, United States, from 2015 to 2019. Prediction accuracies for individual traits varied from 0.23 to 0.79 using different models, with multi-trait models performing superior to uni-trait models for the majority of the traits and validation scenarios.

Seven out of the 14 end-use quality traits from our previous study were selected for multi-trait and multi-environment predictions, which showed lower prediction accuracies and higher genotype by environment interactions (Aoun et al., 2021a; Sandhu et al., 2021c). These higher values of the genotype by environment interactions demonstrated the potential of using multi-trait multi-location models in the breeding programs. We observed a change in genotypes ranking across the multiple environments for these seven traits due to high genotype by environment interactions and negative correlation among the environments. Multi-trait models performed 5.5 and 7.9% superior to uni-trait GS models for within-environment and across-location predictions, while multi-trait multi-environment models performed 10.5% superior to uni-trait GS models. Across-location prediction accuracies for the seven traits varied from 0.23 to 0.53, which were higher than those of previous studies for across-location predictions for end-use quality traits (Lado et al., 2013; Hayes et al., 2017). This was attributed to the reference population, which included the progeny of different lines from the same breeding program. Likewise, Heffner et al. (2011) showed higher across-location prediction for end-use quality by using the same set of biparental populations across the locations. The high prediction accuracy in their study was reflected from a biparental population where training and testing sets must have a relationship and with little variation (Heffner et al., 2011). Furthermore, we observed that genotype by environment interaction components could improve across-location prediction accuracies in the models. Similar work was shown by Ward et al. (2019) and Monteverde et al. (2019) that describe the advantage of including genotype by environment and marker by environment interaction components into the models when correlation among environments is lower.

Predicting breeding values of un-phenotyped individuals is always a daunting task, but different strategies have been employed in recent years for predictions under different circumstances. Inclusion of correlated traits into multi-trait models has been effective to increase predictions for primary traits with low heritability when the secondary trait is highly correlated with high heritability. However, some studies have shown no improvement of prediction accuracies when secondary correlated traits were included into the models for predicting traits in rice (*Oryza sativa* L.) (Schulthess et al., 2016), avocado (He et al., 2016), and mice (Jiang et al., 2015), which could be attributed to some environmental changes or interactions not captured by the associated models. Similarly, Jia and Jannink (2012) showed no advantage of using multi-trait GS models even when traits have high heritability differences. However, in our study, we observed that even though traits have moderate to high heritability, they still showed an increase in prediction accuracies using multi-trait models when the traits have moderate to high correlation. Highest improvement was observed for traits like GPC, FPROT, and FSDS due to their high correlation, whereas CODI showed lowest improvement due to low correlation with other traits. Correlated traits help predict correlated responses when traits of interest are not phenotyped; this will also help predict expensive to phenotype traits. Previous works have shown that prediction accuracies increase when traits have high correlation, but not with low to intermediate correlation among traits (Rutkoski et al., 2012; Jiang et al., 2015).

Our study showed that uni-trait– and multi-trait–based machine and deep learning models performed superior to traditional GS models. We observed that machine and deep learning models performed 5–11% superior to Bayes B and GBLUP under cross-validation and across-location predictions. Liu et al. (2019), Sandhu et al. (2021a) and Zingaretti et al. (2020) also demonstrated the advantage of using deep learning models in soybean (*Glycine* max L.), wheat, and strawberries (*Fragaris ananassa*) over the traditional mixed model–based approaches and supported our findings. Similarly, Montesinos-López et al. (2018) demonstrated the multi-trait–based deep learning model’s superiority over the multi-trait Bayesian models for predicting four different traits in wheat and maize (*Zea mays* L.). These machine learning models are highly flexible for understanding complex interactions present in these datasets, thus inferring the current trends in the datasets compared to parametric models like GBLUP and Bayes B. Furthermore, multi-trait machine learning models are more suitable as they could further explore relationships between traits and sets of predictors with the removal of redundant information from the models with explicit programming. Due to these characteristics of machine and deep learning models, we observed their better performances under uni- and multi-trait scenarios than under Bayes B and GBLUP.

As discussed, multi-trait machine and deep learning models performed better than multi-trait Bayes B and GBLUP models; however, the advantage of machine and deep learning models diminishes when the genotype by environment interaction component was included in the BMTME model. The inclusion of genotype by environment components perfectly models the environmental effects and correlation among the traits for different environments, resulting in improvement of prediction accuracy. Similarly, Guo et al. (2020) and Ibba et al. (2020) showed an increase in prediction accuracies for yield-related traits in U.S. soft wheat and end-use quality traits using multi-trait multi-environment models over the uni-trait models. The comparable performance of multi-trait machine learning models and BMTME models could be attributed to the capacity of BMTME models to provide separate penalization for the genotypes, environment, and genotype by environmental interaction, while working of the machine and deep learning models follow the black-box nature, creating problem for biological understanding of the process.

## Conclusion

We explored the potential of using multi-trait–based genomic selection models for predicting seven end-use quality traits in soft white wheat population. Uni-trait– and multi-trait–based genomic selection models were optimized separately for each trait, and optimized hyperparameters were used for testing. Different cross-validation, independent, and across-location prediction scenarios were applied to compare the model’s performance. Multi-trait genomic selection models performed superior to uni-trait models when traits were correlated with each other. The inclusion of genotype by environment interaction components further improves the across-location prediction accuracies, a typical advantage shown by machine and deep learning models. Prediction accuracies obtained in this study using multi-trait models for within-environment and across-location predictions open up the avenue to explore the use of genomic selection to select for end-use quality traits in wheat. The prediction accuracies obtained in this study further provide evidence of the usefulness of genomic selection in wheat breeding and will enhance the confidence of the breeder to utilize this tool when making selections.

## Data Availability

The datasets presented in this study can be found in online repositories. The names of the repository/repositories and accession number(s) can be found in the article/Supplementary Material.
