# Supplementary material for: Multi-Trait Multi-Environment Genomic Prediction for End-Use Quality Traits in Winter Wheat
Source: Front Genet. 2022 Jan 31;13:831020. doi: 10.3389/fgene.2022.831020 (PMC8841657; doi:10.3389/fgene.2022.831020)
Supplement: Supplementary file 1 [file DataSheet1.docx]

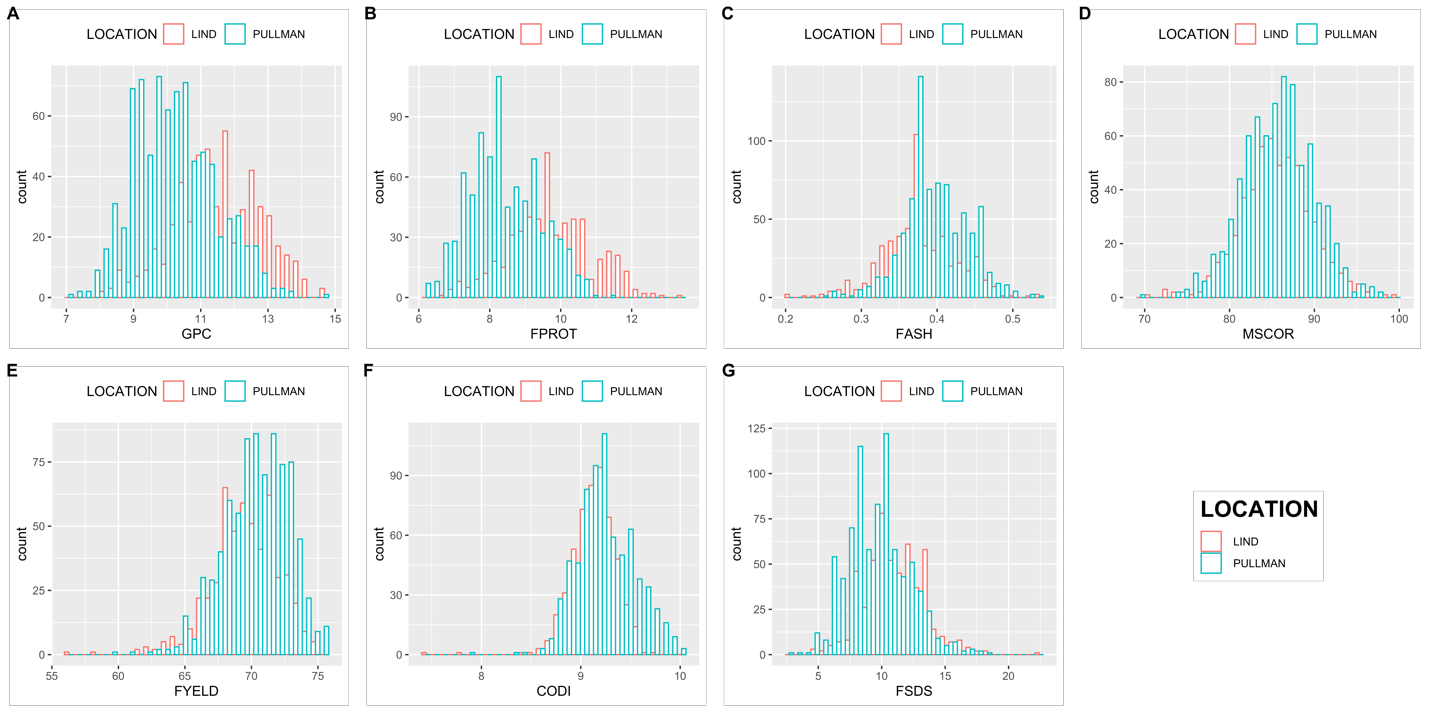


**Supplementary Figure 1.** Frequency distribution of seven end-use quality traits at two locations, namely, Lind and Pullman, WA, USA.

| **Supplementary Table 1.** ANOVA results for the seven end-use quality traits. | | | | | | | | | | | | | | |
| --- | --- | --- | --- | --- | --- | --- | --- | --- | --- | --- | --- | --- | --- | --- |
|  | **GPC** | | **FPROT** | | **FASH** | | **MSCOR** | | **FYELD** | | **CODI** | | **FSDS** | |
|  | F value | P value | F value | P value | F value | P value | F value | P value | F value | P value | F value | P value | F value | P value |
| Genotype | 1.8753 | 8.655e-14*** | 2.1668 | < 2e-16 *** | 2.5213 | <2e-16 *** | 2.4639 | <2e-16 *** | 1.9755 | 9.07e-16 *** | 1.7704 | 6.306e-11 *** | 3.2702 | < 2.2e-16 *** |
| Environment | 230.4984 | < 2.2e-16*** | 270.1156 | < 2e-16 *** | 43.8485 | <2e-16 *** | 33.6539 | <2e-16 *** | 12.6397 | 0.0004154 *** | 38.8867 | 1.072e-09 *** | 60.3476 | 4.945e-14 *** |
| GXE | 4.80 | 0.0082** | 1.3111 | 0.03861* | 3.94 | 0.0042** | 1.04 | 0.021* | 3.40 | 0.01* | 0.1619 | 0.2629 | 1.29 | 0.03* |
| Significance codes: 0 ‘***’ 0.001 ‘**’ 0.01 ‘*’ 0.05 ‘.’ 0.1 ‘ ’ 1 | | | | | | | | | | | | | | |
